# SO_3_H-functionalized carbon fibers for the catalytic transformation of glycerol to glycerol tert-butyl ethers

**DOI:** 10.1038/s41598-023-27432-7

**Published:** 2023-01-11

**Authors:** Karolina Ptaszyńska, Anna Malaika, Magdalena Kapska, Mieczysław Kozłowski

**Affiliations:** Faculty of Chemistry, Adam Mickiewicz University, Uniwersytetu Poznańskiego 8, 61-614 Poznań, Poland

**Keywords:** Chemistry, Materials science

## Abstract

Carbon fibers (CFs) of high quality were produced from hydrocarbons such as isobutane or ethylene using the catalytic chemical vapor deposition method (CCVD) and Ni catalyst. The as-prepared samples were functionalized with acidic groups using concentrated sulfuric acid or 4-benzenediazonium sulfonate (BDS) generated in situ from sulfanilic acid and sodium nitrite. The morphological features of the materials were confirmed by transmission electron microscopy, whereas their physicochemical properties were characterized by means of elemental and textural analyses, thermogravimetric (TG) method, Raman spectroscopy, potentiometric back titration, and X-ray diffraction analysis. The obtained CFs were used as catalysts in glycerol etherification with tert-butyl alcohol at 110 °C under autogenous pressure. The BDS-modified CFs were particularly effective in the reaction, showing high glycerol conversions (of about 45–55% after 6 h) and substantial yields of mono- and di-glycerol ethers. It was found that the chemistry of the sample surface was crucial for the process. The high concentration of -SO_3_H groups decorating CFs boosted the formation of di- and tri-tert-butyl glycerol ethers. Surface oxygen functionalities also had a positive effect on the reaction, however, their impact on the catalytic performances of CFs was significantly weaker compared to that shown by -SO_3_H groups and it was probably due to the adsorption of reagents on the catalyst surface.

## Introduction

Dangerous global climate changes resulting from greenhouse gas emissions have been troubling the world for years. Among the main anthropogenic sources of air pollutants are emissions from the fossil fuel-based industry and the transportation sector^1,2^. Combustion of coal, oil, and gas provides about 84% of global primary energy^3^. However, these non-renewable energy sources are significant providers of hazardous CO_2_, NO_x_, SO_x_, or particulate matter (PM) released into the atmosphere^2,4,5^. Therefore, the development of clean and renewable energy resources is highly justified^1,6^.

The use of wind, solar, and geothermal energy, or hydropower can significantly limit climate changes^1,7^. Unfortunately, the main limitations of renewable-energy power plants are special weather conditions needed for operation, such as wind force or sunlight, the maintenance of which is not always possible^1^. Biomass is another sustainable energy source of large versatility, e.g., it can be easily converted into liquid fuels such as biodiesel. According to the data provided by the International Energy Agency (IEA), global biodiesel production was about 43 billion L in 2021. This value is expected to be maintained in the nearest future^8^.

The process of biodiesel production is accompanied by the release of large quantities of a by-product in the form of (bio)glycerol. It is estimated that for every 1 tonne of biodiesel, 100 kg of glycerol is co-formed^9^. Thus, to make biodiesel fuel more profitable and more competitive with traditional petroleum-based fuels, there is an urgent need to valorize the co-produced glycerol, preferably by converting it into industrially valuable chemicals^9,10^.

Tert-butyl glycerol ethers, TBGE, especially di- and tri-substituted products (DTBGE and TTBGE, respectively), are one of the most attractive compounds obtained from glycerol. These high-substituted glycerol derivatives (h-glycerol ethers) can be widely used in the fuel sector, where they can serve as valuable fuel oxygenates reducing the emissions of harmful gases into the atmosphere during fuel combustion (diesel, biodiesel, or gasoline). Glycerol ethers also improve viscosity, cloud points, or pour points of biodiesel, showing a positive effect on the performance properties of this fuel. Importantly, DTBGE and TTBGE are excellent alternatives to another popular fuel additive, i.e., methyl tert-butyl ether (MTBE), which is considered an environmentally troublesome chemical substance with limited usage in some countries^11–14^.

Typically, tert-butyl glycerol ethers are obtained by the catalytic etherification of glycerol with tert-butyl alcohol (TBA) or isobutene (IB). Despite the lower efficiency of the process with TBA, the use of alcohol is more practical, as IB is more expensive, non-renewable, and requires special reaction conditions to keep the gas in the liquid phase^15,16^.

**Figure 1:**
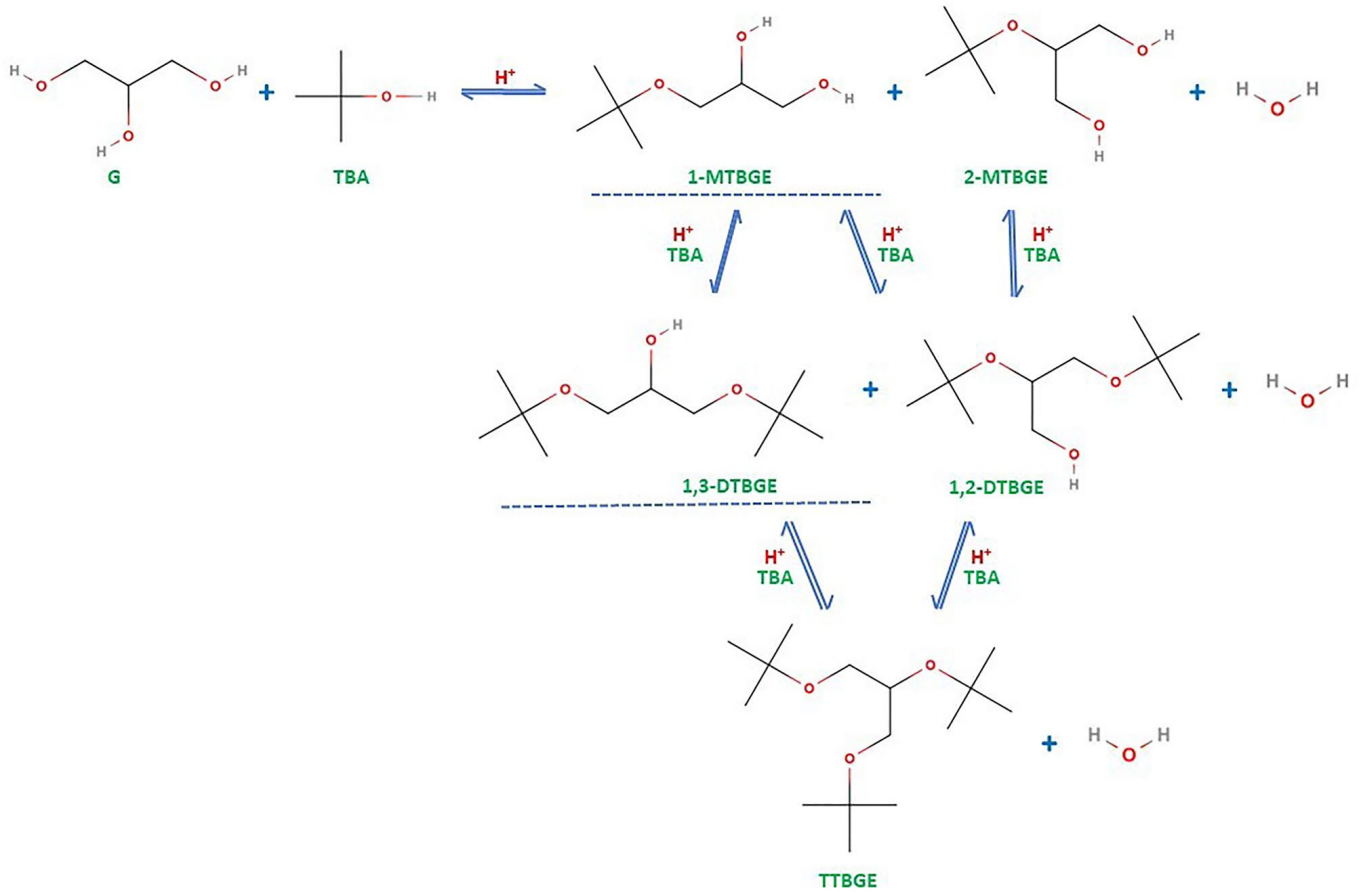
The scheme of reaction between glycerol and tert-butyl alcohol under acidic conditions.

Conversion of glycerol to TBGE is an acid-catalyzed reaction in which heterogeneous catalysts are preferred over homogeneous ones. Solids have several advantages, e.g., they can be easily separated from the reaction mixture, are not corrosive, and are more environmentally friendly than homogeneous catalysts^11,12,17^. The scheme of reaction between glycerol and TBA (prepared according to a commonly accepted mechanism) is shown in Fig. 1. As can be seen, five different glycerol tert-butyl ethers can be formed. Importantly, due to the steric effects (a tert-butyl group is of voluminous size), the formation of primary ethers (i.e., 1-substituted and 1,3-substituted) is favored^18^.

Ion exchange resins, modified zeolites, and silicas are solid catalysts most frequently tested in glycerol etherification, which is due to their high activities^12,19–22^. For instance, Melero et al.^21^ demonstrated that sulfonic acid-functionalized mesostructured silicas showed excellent catalytic behavior in the etherification of glycerol with IB, giving glycerol conversions up to 100% and combined selectivities toward DTBGE and TTBGE over 92% within 4 h. Frusteri et al.^23^ tested spherical silica-supported Hyflon® catalysts (SSHC) in the process between glycerol and IB and found that the best catalytic system led to complete conversion of glycerol and high combined selectivity to the desired ethers (i.e., DTBGE and TTBGE), of about 97%, just within 6 h. The catalyst also showed high resistance to deactivation. Xiao et al.^20^ investigated glycerol etherification with IB over HY zeolites modified with citric acid and achieved glycerol conversion and a combined selectivity to di- and tri-tert-butyl glycerol ethers of 91% and 64%, respectively, within 7 h of the reaction. On the other hand, Klepáčová et al.^18^ performed glycerol etherification with tert-butyl alcohol using Amberlyst-15 and large-porous zeolites such as H-BEA. The conversions of glycerol were comparable for both samples (> 80 and 90% after 6 h); however, H-BEA formed nearly twice as much di-ethers as Amberlyst-15 (45% vs. 25%).

Carbon materials have been relatively rarely used in glycerol etherification. Meanwhile, these catalysts offer several advantages over typical catalytic systems, e.g., high thermal stability, ease of tuning their physicochemical properties, and various morphological forms^17,24^. For instance, Zhao et al.^11^ obtained very good catalytic results in glycerol etherification performed over a sulfonated carbon catalyst synthesized from peanut shells, i.e., complete conversion of glycerol and a 92.1% combined selectivity to DTBGE and TTBGE just within 2 h; however, the process used isobutylene which presents several disadvantages compared to TBA. Gonçalves et al.^25^ tested sulfonated carbon-based catalysts in the etherification of glycerol with TBA. Over 80% glycerol conversion and about 21% selectivity to DTBGE and TTBGE were achieved after 4 h of the reaction at 120 °C. The catalytic activity of the prepared carbon samples was ascribed mainly to the presence of strongly acidic surface sulfonic groups; however, no detailed studies on the influence of the carbon catalyst properties on the material activity have been performed. In turn, Estevez et al.^26^ demonstrated that sulfonated carbon obtained from olive stones gave a 21% yield of higher glycerol ethers just within 15 min. Additionally, the prepared catalyst exhibited high stability, maintaining its activity after being used in several consecutive reactions. However, the process required a microwave to achieve these promising results.

The current work presents the results on the catalytic performances of the SO_3_H-functionalized carbon fibers produced from isobutane or ethylene in glycerol etherification carried out with sustainable and easy-to-handle TBA. Importantly, the collected data enabled us to determine the relationship between the chemical structure and the carbon fibers’ performance in the formation of di- and tri-glycerol ethers (DTBGE and TTBGE, respectively), i.e., the products of interest. We believe that these findings can set the direction of future research aimed at maximizing the production of h-glycerol ethers and at developing new effective acid carbon catalysts for the glycerol valorization processes. Furthermore, to the best of our knowledge, modified ethylene- and isobutane-derived CF catalysts have never been tested in TBA-assisted etherification of glycerol.

## Experimental section

### Preparation of the carbon samples

Carbon fibers (CFs) were prepared by catalytic chemical vapor deposition (CCVD) using gaseous hydrocarbons such as isobutane or ethylene as carbon feedstocks, and nickel as a catalyst. The process was conducted under the optimized conditions established earlier^27,28^. Briefly, NiO (100 mg in the case of isobutane and 30 mg in the case of ethylene) was placed in a quartz boat in a horizontal tube furnace and heated under Ar flow to 550 °C. At this temperature, the reduction of Ni oxide was performed using a 20%H_2_/80%Ar mixture (total flow of gases of 100 cm^3^/min) for 2 h. Afterward, the CCVD process of hydrocarbons was initiated by switching the H_2_/Ar gases to a mixture of 75%H_2_/25%ethylene or 50%H_2_/50%isobutane. The first reaction was performed at 550 °C, whereas the second at 600 °C, both for 4 h. In both cases, the obtained carbon product was treated with a 21% HCl solution and heated for 2 h under reflux to remove the Ni catalyst from the sample. Afterward, the material was washed with hot distilled water, dried at 110 °C overnight, and finally, sieved to a particle size ≤ 0.4 mm. The resultant samples were denoted as CF_i-bu_ and CF_et_ depending on the carbon precursor used in the CCVD process (i.e., carbon fibers from isobutane or ethylene, respectively).

The prepared CF samples were functionalized with acidic sites using sulfuric acid or 4-benzenediazonium sulfonate (BDS) generated in situ. Details of the modifications are given below.

The modification of CFs with sulfuric acid was carried out by mixing 3.5 g of the sample with 90 cm^3^ of concentrated H_2_SO_4_. The mixture was heated at 140 °C for 20 h under argon flow. After the functionalization, the carbon was filtered out, thoroughly washed with hot distilled water until a neutral pH, and dried overnight at 110 °C. Finally, it was sieved to a particle size of ≤ 0.4 mm. The resultant CF samples were labeled as CF_i-bu_-H_2_SO_4_ and CF_et_-H_2_SO_4_.

The reaction of CFs with BDS was carried out according to a modified procedure proposed originally by Toupin and Bélanger^29^. The process was performed using 3.5 g of the carbon that was mixed with distilled water (175 cm^3^), sulfanilic acid (5 g), and sodium nitrite (2 g). In the next step, concentrated hydrochloric acid (35 cm^3^) was added dropwise to the mixture. The modification was performed at 20 °C for 20 h. Afterward, the sample was washed with distilled water, then with methanol, dimethylformamide (DMF), and acetone. Finally, it was dried overnight at 110 °C and sieved to a particle size of ≤ 0.4 mm. The resultant samples were denoted as CF_i-bu_-BDS and CF_et_-BDS.

### Characterization of the samples

The obtained carbon samples were characterized using various techniques, i.e., elemental and textural analyses, electron microscopy, potentiometric back titration method, Raman spectroscopy, thermogravimetric analysis, and XRD technique.

The elemental CHNS composition of the samples was determined using an elemental analyzer Vario EL III. Textural parameters of the carbons were examined by means of a Quantachrome Autosorb IQ apparatus based on N_2_ adsorption/desorption measurements performed at -196 °C. The samples were outgassed under vacuum conditions at 150 °C before the analysis. Apparent surface areas of the samples (S_BET_) were calculated using the Brunauer–Emmett–Teller (BET) model, while the micropore volumes (V_micro_) and external surface areas (of meso- and macropores; S_ext_) were determined using the t-plot method. In turn, the total volumes of pores (V_tot_) were calculated from the amount of nitrogen adsorbed at a relative pressure close to 1. A potentiometric back titration method was used to measure the total acidity of the prepared carbons. For this purpose, a Cerko Lab microtitration unit was applied. The measurement procedure included mixing a sample (100 mg) with a 0.01 M NaOH solution (50 cm^3^) and shaking the obtained suspension at room temperature for 20 h. Afterward, the mixture was filtered and a clear solution was titrated with 0.05 M HCl. The morphological features of the prepared materials were examined using a TEM JEOL 2000 transmission electron microscope operating at 80 kV. In turn, high-resolution TEM (HR-TEM) images were obtained by means of an FEI Tecnai G2 20 X-TWIN apparatus. A Bruker AXS D8 Advance diffractometer was applied to perform X-ray diffraction (XRD) measurements. Raman spectra were acquired using a Renishaw InVia Reflex confocal Raman spectrometer equipped with an argon laser as an excitation source (λ = 514 nm, P = 1 mW). In turn, thermogravimetric analysis (TG) was performed using a Setaram Setsys 1200 thermal analyzer in nitrogen or air flow in the temperature range of 20–950 °C and with a heating rate of 10 °C/min.

### Catalytic measurements

Etherification of glycerol was carried out in a hand-made high-pressure stainless steel laboratory autoclave. This model consisted of a reactor body, a thermocouple inserted into the reaction mixture to precisely control the process temperature, an inlet gas valve for purging the reactor, a pressure gauge to monitor the current reaction pressure, a heating jacket, a magnetic stirrer, and a sampling capillary with an outlet valve enabling in-situ collecting the samples. The reactor was charged with 10.2 g of glycerol (G) and 42 cm^3^ of tert-butyl alcohol (TBA; TBA:G molar ratio of 4:1). Afterward, the catalyst was added to the mixture (5 wt.% based on the glycerol weight). To remove air from the reactor, the autoclave was flushed with argon several times. The etherification process was performed at 110 °C for 24 h under autogenous pressure. To monitor the progress of reaction, samples of the reaction mixtures were taken after 1, 2, 4, 6, and 24 h. The used sampling procedure did not change notably the pressure inside the reactor.

The analysis of the liquid samples was performed with the use of a gas chromatograph (SRI 8610C) equipped with a RESTEK MXT®—WAX capillary column (30 m × 0.25 mm × 0.25 μm) and a flame ionization detector (FID) working at the temperature of 210 °C and powered by hydrogen and air with flows of about 25 cm^3^/min and 270 cm^3^/min, respectively. Helium was used as the carrier gas (1 cm^3^/min) and a split injector (temperature of 230 °C and a split ratio of about 30) was applied for the analyses. Measurements were carried out according to the following temperature program: 40 °C (7 min) and 210 °C (17 min; increase rate of 30 °C/min). The identification of the reaction components was confirmed by a GC–MS (Gas Chromatography-Mass Spectrometry) technique.

The activity of catalysts was expressed as conversions of glycerol (X_G_) and yields of the corresponding glycerol ethers (i.e., Y_MTBGE_—yield of MTBGE, Y_DTBGE_—yield of DTBGE, and Y_TTBGE_—yield of TTBGE), or selectivity to individual reaction products (i.e., S_MTBGE_—selectivity to MTBGE, S_DTBGE_—selectivity to DTBGE, and S_TTBGE_—selectivity to TTBGE).

## Results and discussion

The calculated yields of the carbon fibers obtained from ethylene and isobutane (both used in a mixture with hydrogen; see Experimental) were about 143 and 52 gCFs/gNi, respectively. These values were quite comparable or even significantly higher than those achieved by other authors. For example, Toebes et al.^30^ obtained a yield of CFs of about 20 g/gMe using a C_2_H_4_/H_2_ mixture and Ni as a catalyst. Moreover, the use of ethylene as a carbon source was found to be more advantageous than the use of other hydrocarbons. For instance, according to our previous study^27^, the use of methane in CCVD resulted in a very low yield of carbon nanofibers, i.e., about 4 gCFs/gNi. Similarly, studies by Miniach et al.^31^ showed that the activity of Ni/hydroxyapatite in the CFs formation via CCVD of methane was only about 10 gCFs/gCat. Filamentous carbon was also produced from acetylene—31.6 g of coiled CFs per 1 g of a Fe-based catalyst was obtained in this case^32^. In view of the above, the results of yields obtained in this study seem to be quite promising, even those achieved for CF_i-bu_ produced with lower efficiency.

A thorough characterization of the obtained samples as well as the results of their catalytic performances are presented below.

### Characterization of the samples

The elemental composition of the prepared materials is presented in Table 1. As can be observed, the initial CFs showed a very high content of carbon, which was about 97% in the case of CF_i-bu_ and 99% for CF_et_. Only traces of heteroatoms were detected (the O contents between 0.2 and 0.9%). These results are not surprising as the preparation of CFs involved the use of hydrocarbons as carbon feedstocks, thus the compounds with no oxygen atoms in their structures (traces of O in CFs probably come from the oxidation processes taking place during the sample storage). In the case of functionalized materials, a reduction in the content of elemental carbon was observed, although the C contents in the modified samples were still very high—over 90%. At the same time, an increase in the contents of heteroatoms such as sulfur and oxygen was noticed. This indicates that the methods used here for the modifications of CFs were successful. It should be stressed, however, that the degree of sample functionalization was not very high (the S contents between 0.4 and 1.4% were attained), which is not surprising taking into account a rigid and ordered structure of typical carbon fibers^28,33^. For comparison, our previous studies showed that modifications of carbon xerogels can produce samples with a 1.4—2.3% S content, while functionalization of carbon polymeric spheres can give materials with a sulfur content of 4.3%^34^. In turn, the sulfonated bio-carbons obtained in the process of simultaneous partial carbonization and sulfonation contained 0.7—5.0% of sulfur in their structures^35^.

**Table 1:** Results of the elemental analysis (wt.%), total acidity measurements (A_tot_, mmol H^+^/g), sulfonic groups (–SO_3_H, mmol/g) and ash content determinations for the obtained samples.

Sample	Ash	C	H	N	S	O*	–SO_3_H^#^	A_tot_
CF_i-bu_	0.3	99.0	0.4	0.1	0.0	0.2	0.00	0.00
CF_i-bu_-H_2_SO_4_	0.5	96.4	0.7	0.0	0.4	2.0	0.11	0.09
CF_i-bu_-BDS	0.8	94.6	0.8	0.3	0.9	2.6	0.27	0.01
CF_et_	1.1	97.3	0.7	0.0	0.0	0.9	0.00	0.03
CF_et_-H_2_SO_4_	0.5	92.2	0.7	0.1	0.6	5.9	0.17	0.39
CF_et_-BDS	0.8	91.0	1.1	0.5	1.4	5.2	0.42	0.07

*Calculated by difference; ^#^calculated from the elemental analysis.

The functionalization degree of the CF samples varied depending on the modifying approach used. In the case of CFs modified with sulfuric acid, the amount of S was 0.4% and 0.6% (for CF_i-bu_-H_2_SO_4_ and CF_et_-BDS, respectively), while the reaction of CFs with diazonium salt introduced about twice as much sulfur into the CF structure (see Table 1). Importantly, sulfur in the BDS-modified carbons was probably anchored to the sample surface in the form of -PhSO_3_H groups, which was also suggested by literature data^36,37^. For the BDS-modified carbons, there was a noticeable increase in the content of nitrogen. This may indicate the presence of azo bonds in the prepared materials, which were formed in coupling reactions between diazonium cations and the surface of carbons^29^.

Generally, the modifications of CFs with H_2_SO_4_ turned out to be less effective than those with BDS. This is also in line with literature data indicating lower susceptibility of graphite-like materials to sulfonation with concentrated sulfuric acid compared to that of amorphous ones^38^. As depicted in Table 1, the unmodified carbons practically did not show acidic properties as A_tot_ measured for CF_i-bu_ and CF_et_ was very low. Generally, the used modifications increased A_tot_; however, the changes between the functionalized and parent CFs were not so significant, especially in the case of CF_i-bu_-H_2_SO_4_ and for BDS-modified samples. Interestingly, the total acidities measured for BDS-modified CFs were lower than those resulting from the number of surface sulfonic groups (calculated based on the sulfur content). This may be related to the neutralization of some -SO_3_H structures, e.g., by the formation of zwitterions and finally, the creation of SO_3_^-^ moieties on the carbon surface^36,39–41^. The opposite effect was observed for CF_et_-H_2_SO_4_, i.e., the total acidity measured for this sample was higher than that resulted from the presence of -SO_3_H groups. This can indicate the formation of surface oxygen groups of acidic properties during the CF_et_ reaction with H_2_SO_4_ apart from the sulfonic functionalities (see also the oxygen contents in CF_et_-H_2_SO_4_ and CF_et_ in Table 1). This is in accordance with previous literature reports suggesting oxidizing properties of concentrated sulfuric acid^41–43^. The surface of CF_i-bu_ seemed to be more resistant to oxidation (as -SO_3_H ≈ A_tot_) as well as the functionalization in general (CF_i-bu_ was functionalized less effectively compared to CF_et_).

Table 2 presents the textural properties of the obtained samples. The unmodified CFs showed moderate apparent surface areas (S_BET_) which differed slightly depending on the carbon precursor used during the CCVD process. Both types of the initial samples showed a significant contribution of the external surface area to the apparent surface area (S_ext_/S_BET_), and only a small number of micropores was detected in both cases (as evidenced by a low contribution of the micropore volume to the total volume of pores; V_micro_/V_tot_). The modifications of CFs with sulfuric acid caused only slight decreases in S_BET_, while the reactions with BDS resulted in more significant S_BET_ changes. This was probably due to introducing high volume -PhSO_3_H groups into the structure of CFs during the reaction with BDS, blocking the smallest pores in the samples (as V_micro_/V_tot_ for both BDS-treated samples decreased to 0%). A similar phenomenon was also observed elsewhere^35,44^. Interestingly, the observed decrease in S_BET_ was higher in the case of CF_et_-BDS, suggesting more effective functionalization of this sample compared to CF_i-bu_-BDS. This conclusion is also in line with the results of elemental analysis (see also the contents of S in Table 1).

**Table 2:** Textural analysis of the samples.

Sample	S_BET_ (m^2^/g)	S_ext_/S_BET_*100 (%)	V_micro_/V_tot_*100 (%)
CF_i-bu_	115	59	8.93
CF_i-bu_-H_2_SO_4_	102	58	9.62
CF_i-bu_-BDS	42	100	0.00
CF_et_	174	62	8.63
CF_et_-H_2_SO_4_	171	67	8.76
CF_et_-BDS	58	100	0.00

**Figure 2:**
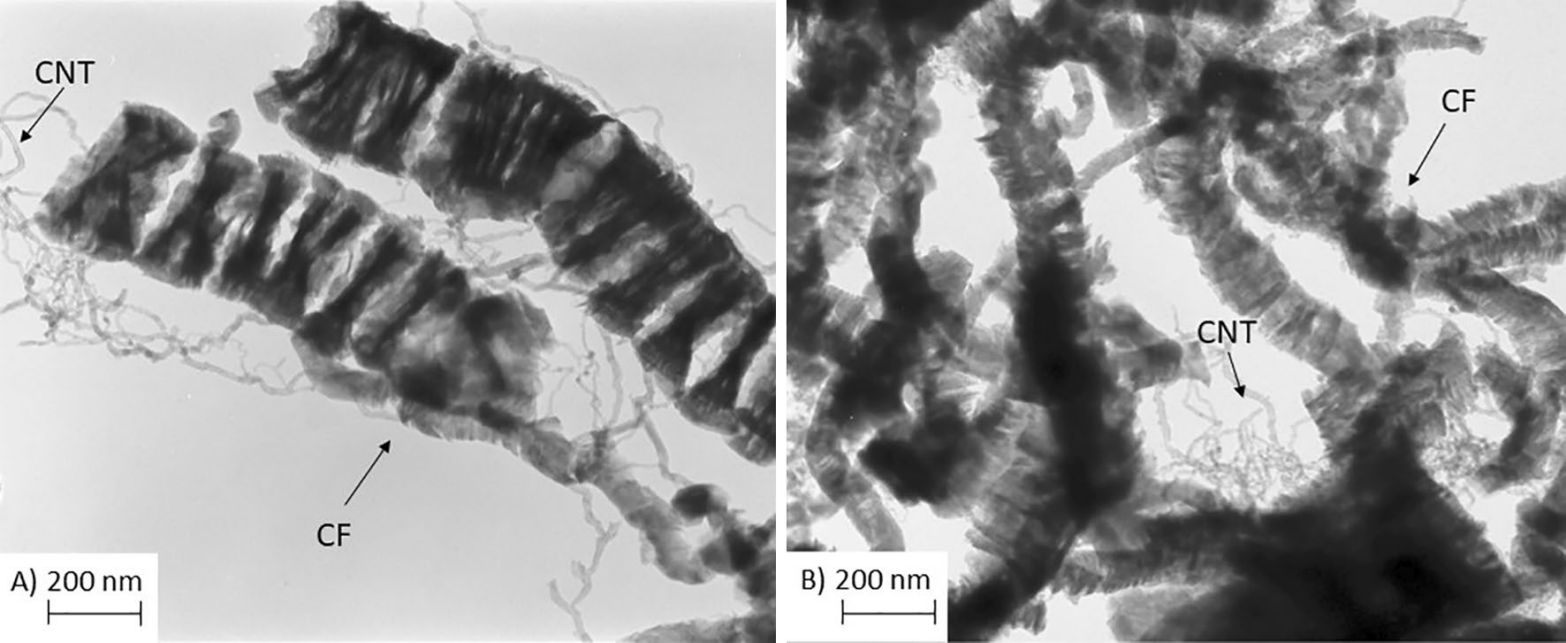
TEM micrographs of (**A**) CF_i-bu_ and (**B**) CF_et_.

The morphology of selected carbon samples prepared via the CCVD method is presented in Fig. 2. As can be observed from Fig. 2A, the process with the use of isobutane and Ni as catalyst led to a mixture of different carbon structures, differing in their sizes and shapes. Among them, mostly carbon fibers were identified—rather short, with a fairly large diameter (marked as CF in the TEM image). Probably, also carbon nanotubes with small diameters were formed in the process, as long fine hollow structures are visible in Fig. 2A (marked as CNT).

Figure 2B shows a TEM image of the sample produced from ethylene. Similarly to CF_i-bu_, CF_et_ consisted of carbon fibers with rather large diameters (however, slightly smaller than in the case of CF_i-bu_). Individual filaments showed irregular shapes and surfaces—from smooth to presenting sharp edges. Trace amounts of CNT are also visible.

The high-resolution TEM images of different magnifications obtained for CFs produced from isobutane and ethylene are shown in Fig. 1SM.

The presented pictures revealed the partial graphitic structure of CF_i-bu_ and CF_et_, as the ordered orientation of the graphene sheets was visible for most of the produced fibers. In the case of CFs obtained from ethylene, the graphene sheets were arranged parallel to each other and perpendicular to the fiber axis, forming platelet carbon fibers (Fig. 1SM E). CFs produced from isobutane also resembled platelet-like structures, however, a slightly lower degree of order than that observed for CF_et_ was noticed, as randomly oriented graphene sheets were also found in the HR-TEM images of CF_i-bu_ (Fig. 1SM B).

**Figure 3:**
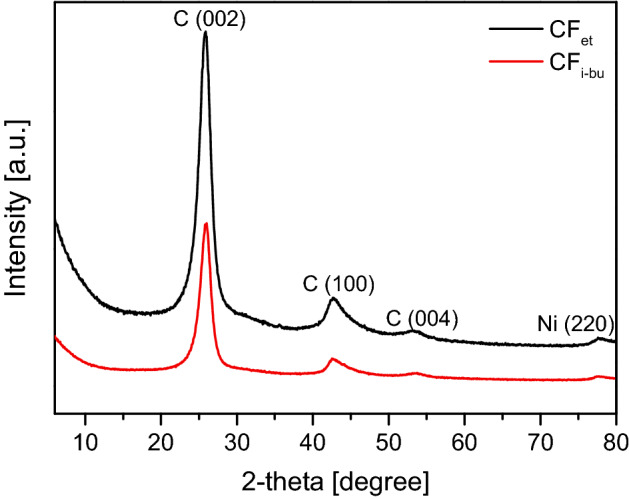
The XRD patterns of the prepared initial CF samples.

The XRD patterns of the unmodified carbon fibers (i.e., CF_et_ and CF_i-bu_) are shown in Fig. 3. In both cases, two signals characteristic of carbon materials and attributed to graphite-like structures are visible—a sharp diffraction peak at 2-theta of 26° and a less intense one at 2-theta of 43°, which corresponds to diffraction on the (002) and (100) planes, respectively. A weak signal at 2-theta of 55° is also noticeable, probably representing the (004) graphite-type reflection^45–49^. The XRD patterns also show a diffraction line at 2-theta of 78°, possibly belonging to the (110) plane of graphitic materials^50^ or metallic nickel^27,51^. The presence of this peak can suggest that some traces of catalyst remained in the carbon product despite the sample boiling with a solution of HCl (see Experimental).

**Figure 4:**
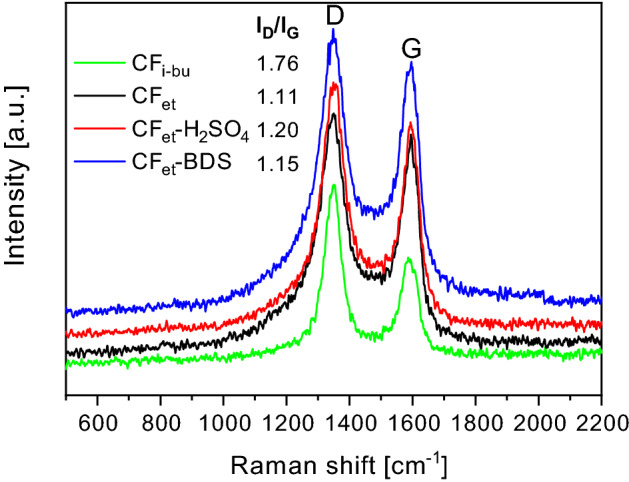
Raman spectra of selected samples.

Figure 4 presents the Raman spectra obtained for the CFs produced from different precursors as well as the data achieved for a series of modified carbons. As can be observed, all the samples showed peaks typical for carbon materials, i.e., at about 1350 and 1580 cm^-1^. The first signal (D band) is typically attributed to defects and disorders in the lattice structure of carbons, while the latter (G band) is related to the graphitized carbon motifs where the C-atoms are sp^2^ hybridized. Importantly, the relative intensity ratio of the D to G band (I_D_/I_G_) can serve as an indicator of carbon sample graphitization and simultaneously as an indicator of structural defects of carbons^52^.

As shown in Fig. 4, the I_D_/I_G_ value calculated for CFs produced from ethylene was significantly lower than that of CFs synthesized from isobutane (~ 1.11 vs. ~ 1.76), implying a higher degree of CF_et_ graphitization and its less defective nature. This conclusion is also in line with the results achieved in the HR-TEM analysis (see Fig. 1SM). The modified carbon fibers showed slightly higher values of I_D_/I_G_ ratios as compared to the parent sample, which indicates that some defects at the sidewalls of the fibers appeared after the functionalizations. It might be supposed that these defects were due to the carbon sp^2^ to sp^3^ hybridization, being a result of the binding of new functional groups to the carbon surface (see also the results of EA in Table 1)^53^ As sulfuric acid is a more aggressive reagent than BDS and additionally shows oxidizing properties (as discussed earlier), CF_et_-H_2_SO_4_ was found to be slightly more defective than CF_et_-BDS, which was suggested by a slightly higher value of I_D_/I_G_ obtained for CF_et_-H_2_SO_4_ compared to that achieved for CF_et_-BDS.

**Figure 5:**
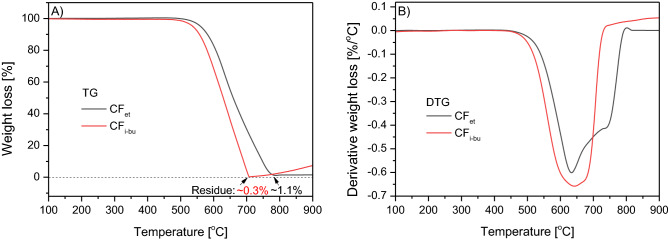
TG (**A**) and DTG (**B**) curves of the CF_et_ and CF_i-bu_ samples (air flow).

Figure 5 shows the results of thermogravimetric (TG) analysis of CF_et_ and CF_i-bu_ samples performed in an air atmosphere. As can be seen from the TG pattern (Fig. 5A), both samples were thermally stable up to about 500 °C. Over this temperature, a sharp weight loss was detected in both cases. The complete combustion of the materials was noted at 700 °C and 780 °C. The residue after combustion was 0.3% and 1.1% for CF_i-bu_ and CF_et_, respectively, and its presence was probably related to Ni remaining in the samples after the CCVD process. Under the measurement conditions, a slight weight gain was also observed at high temperatures as a result of the metal oxidation^54^. As shown earlier, traces of Ni in the CF samples were also confirmed by the XRD analysis (see also Fig. 3). In general, the DTG patterns of the samples (Fig. 5B) show intense peaks with the minima at a temperature of about 640 °C. These signals can be attributed to the combustion of well-ordered filamentous carbon^27,54–56^. Furthermore, in the DTG curve of CF_et_, an additional poorly separated signal can be distinguished (at about 750 °C). The above observations can suggest the presence of phases with a different number of defects in CF_et_, i.e., a slightly more defective phase (with a minimum at 640 °C), and the one more stable and well-organized (with a minimum at 750 °C)^28,57^. The absence of lower-temperature peaks excludes contamination of the samples with less thermally stable types of carbon, e.g., amorphous carbon (combustion temp. below 400 °C)^27,54,55^.

**Figure 6:**
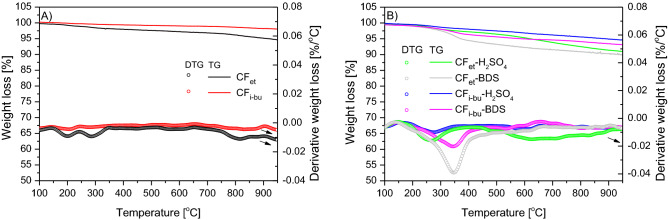
TG and DTG curves of the parent samples (**A**) and the modified CFs (**B**) for the analyses performed in nitrogen flow.

Figure 6 shows the results of the TG analysis performed for the pristine and modified CF_et_ and CF_i-bu_ in nitrogen flow. In the case of unmodified materials (Fig. 6A), only a slight decrease in the sample weight with increasing temperature was observed, which proves the high thermal resistance of the samples tested. At the final temperature of TG measurement, the weight loss was ~ 6% for CF_et_ and ~ 3% for CF_i-bu_. The DTG profiles of the parent carbons show two small peaks with minima at about 200 °C and 280 °C, suggesting the presence of oxygen surface groups (such as carboxylic ones) in CFs. These groups could spontaneously form on the carbon surface during the sample contact with air^58^. The existence of oxygen-type functionalities was also confirmed by elemental analysis (see also Table 1).

Significantly higher weight losses were observed for the modified CF samples (Fig. 6B), reflecting the degree of functionalization of these materials. Only a slight decrease in the sample weight was observed for CF_i-bu_-H_2_SO_4_ (~ 6%), indicating a low number of groups released as volatiles. This is also in line with the results of elemental analysis (see Table 1). On the other hand, the most significant changes in the sample mass were observed for CF_et_-BDS (~ 11%) which was considered the most functionalized material among the prepared modified CFs (see also Table 1). Importantly, the DTG patterns of H_2_SO_4_-modified samples indicate the presence of signals with minima at 280 °C, which can be attributed to the decomposition of surface sulfonic groups^39,42,59^. Moreover, the DTG profile of CF_et_-H_2_SO_4_ depicts a broad peak starting from about 500 °C, which probably is due to the presence of more stable oxygen groups, e.g., phenolic ones, formed during the sample modification with concentrated H_2_SO_4_^39,42,58,59^. A much lower intensity of this peak in the profile of CF_i-bu_-H_2_SO_4_ indicates higher resistance of CF_i-bu_ to oxidation, which was also concluded based on the elemental analysis (Table 1). Different DTG curves were observed for the samples modified with BDS as they both show an intense peak with a minimum at 350 °C. This signal can be ascribed to the decomposition of -PhSO_3_H groups^39,60^. No signals were observed at higher temperatures, which proves that the applied functionalization method did not oxidize the sample.

### Catalytic results

The obtained functionalized CFs were tested as catalysts in the process of glycerol etherification with tert-butyl alcohol. For the sake of comparison, also a blank test and a reaction with a commercial catalyst, Amberlyst-15, were performed.

**Figure 7:**
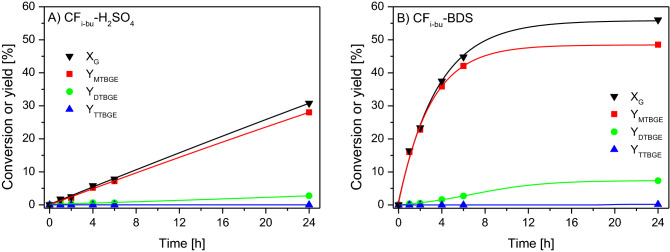
Conversions of glycerol (X_G_) and yields (Y) of individual products obtained in the glycerol etherification performed with tert-butyl alcohol over (**A**) CF_i-bu_-H_2_SO_4_, (**B**) CF_i-bu_-BDS.

**Figure 8:**
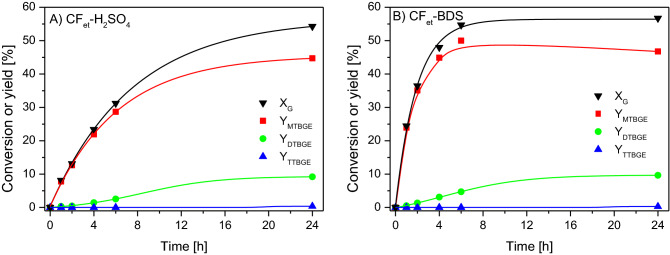
Conversions of glycerol (X_G_) and yields (Y) of individual products obtained in the glycerol etherification performed with tert-butyl alcohol over (**A**) CF_et_-H_2_SO_4_ and (**B**) CF_et_-BDS.

The results obtained in the reaction over unmodified CFs showed that the catalytic effect of the parent samples was negligible as glycerol conversion achieved after 24 h was below 2% in both cases. Much better results were attained for the modified CFs. The catalytic performances of the functionalized materials are gathered in Figs. 7 and 8.

As can be observed, the catalytic properties of H_2_SO_4_- and BDS-modified CF_i-bu_ differed significantly. In the case of CF_i-bu_-H_2_SO_4_ (Fig. 7A), only small amounts of glycerol were reacted at the beginning of the process, i.e., X_G_ after 1 h was only about 2%. As the reaction progressed, this parameter increased to approximately 30% (after 24 h). The yields of individual ethers varied considerably. The dominant products at every stage of the process were mono-substituted ethers (i.e., MTBGE). After 1 h of the reaction, their yield was about 2%. Y_MTBGE_ increased linearly with time—to about 28% after 24 h of the process. The yield of DTBGE was significantly lower and after 24 h, it was less than 3%. A tri-substituted product (i.e., TTBGE) was formed only in traces. Comparing the catalytic activity of CF_i-bu_-H_2_SO_4_ to that of the unmodified sample, it was obvious that the observed catalytic effect was a result of the acidic treatment of CF_i-bu_ and a consequent slight increase in the -SO_3_H content and the sample acidity (see Table 1). However, as the functionalization was not very effective, the obtained catalytic data were not so impressive.

It can be seen in Fig. 7B that CF_i-bu_-BDS catalyzed the reaction quite effectively, as significant amounts of glycerol were transformed into glycerol ethers in the first hours of the process. Over 16% of glycerol was converted within 1 h of the reaction and after 6 h, X_G_ was about 45%. As before, the glycerol conversion increased over time, and finally, it reached about 56%. The yields of individual ethers differed significantly. Mono-substituted products were formed preferentially and the yield of MTBGE after 24 h was about 45%. The DTBGE yield was much lower and it reached just over 7% after 24 h of the process. TTBGE was formed in the smallest amounts (Y_TTBGE_ after 24 h was less than 0.2%).

CFs obtained from various carbon precursors (i.e., isobutane and ethylene) worked differently (compare Figs. 7 and 8). The modified CFs produced from ethylene were generally more active in glycerol etherification than their counterparts produced from isobutane. This was due to the susceptibility of the obtained fibers to functionalization, especially with sulfuric acid, and the chemistry of their surfaces (see Table 1 and discussion in 3.1). As can be observed in Fig. 8A, CF_et_-H_2_SO_4_ transformed glycerol quite effectively. After 1 h, X_G_ was slightly above 8%, and finally, it reached the value of 54%. The MTBGE products were mostly formed and their yield after 1 h of the reaction was about 8%. After 24 h, Y_MTBGE_ increased up to 45%. Di-substituted ethers were formed to a much lesser extent; however, their yield was higher than in the reaction performed in the presence of CF_i-bu_-H_2_SO_4_. TTBGE was again created only in traces. Quite good catalytic results obtained for CF_et_-H_2_SO_4_ can be attributed to the relatively high acidity of this sample (0.39 mmol H^+^/g, see Table 1). On the other hand, it should be stressed that in this case the concentration of -SO_3_H groups was not very high (i.e., 0.17 mmol/g, Table 1) and, as mentioned before, the sample acidity was partially generated also by the presence of oxygen acidic groups (such as phenolic or carboxylic ones^34,41–43^). Thus, it can be supposed that the role of such functionalities in the etherification reaction might also be significant (which will be discussed later).

The CF_et_-BDS sample was more active in the process than its H_2_SO_4_-modified counterpart (especially at the beginning of the reaction), which was probably related to a high concentration of -SO_3_H groups on the carbon surface (0.42 mmol/g, Table 1). As can be seen in Fig. 8B, a 25% glycerol conversion was obtained after 1 h of the reaction. X_G_ after 24 h increased to 57%. Mono-substituted compounds were formed in the highest amounts (Y_MTBGE_ about 25% vs. Y_DTBGE_ ~ 0,5% after 1 h). Interestingly, the MTBGE yield began to drop after 6 h of the process, and finally, it reached about 47%. At the same time, quite a significant increase in Y_DTBGE_ was observed. Probably, this phenomenon was due to the reaction mechanism and transformation of mono-substituted glycerol ethers to di-substituted ones. The yield of TTBGE after 24 h was less than 0.3%.

**Figure 9:**
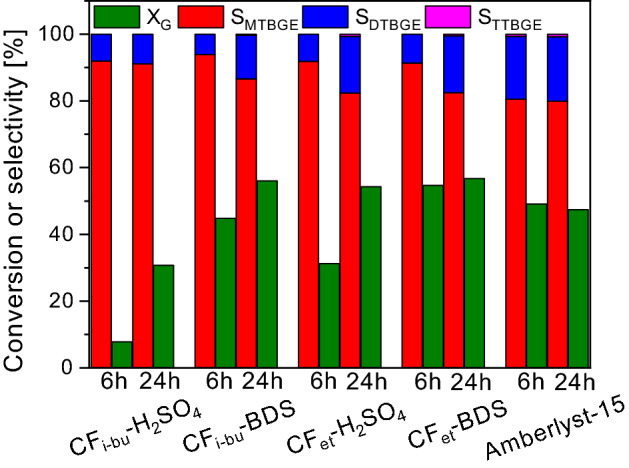
Glycerol conversions and selectivities to individual products after 6 and 24 h of the reaction using the prepared CFs and a commercial catalyst, Amberlyst-15.

Figure 9 presents a comparison of the catalytic performances of all modified CFs to that of a commercial catalyst, Amberlyst-15 (expressed as glycerol conversions and selectivities to individual glycerol ethers after 6 and 24 h).

As can be seen, there was a group of catalysts that worked very effectively in the process, giving a high glycerol conversion and selectivity to di-substituted ethers just within 6 h of the reaction. These samples were both CFs modified with BDS and a commercial catalyst, Amberlyst 15. For these catalysts, an extension of the reaction time to 24 h did not affect significantly the conversion of glycerol as X_G_ changed rather slightly with time. Interestingly, X_G_ obtained after 24 h in the reaction over Amberlyst-15 was even lower than the results achieved with the prepared CFs (apart from CF_i-bu_-H_2_SO_4_). Considering the selectivities to different ethers, MTBGE was the main product in each of the performed processes. However, CFs and Amberlyst-15 worked a little bit differently, i.e., in the case of modified CFs, S_MTBGE_ after 6 h was over 90%, while Amberlyst-15 presented S_MTBGE_ of ~ 80%. On the other hand, S_DTBGE_ obtained in the reaction with the modified CFs did not exceed 10% after 6 h, while in the process with Amberlyst-15, it was almost 19%. This can indicate a faster transformation of mono-substituted ethers to di-substituted products in the case of Amberlyst-15, probably due to the high acidity shown by this catalyst (4.7 mmol/g)^17^. In most cases, the selectivities to MTBGE decreased with time in favor of DTBGE production, which can confirm that the mechanism of glycerol etherification performed with TBA over carbon catalysts includes a series of consecutive reactions (Fig. 1). The selectivity to TTBGE was close to zero in all the discussed processes and did not change significantly with time. Overall, the highest selectivities to the target ethers (i.e., DTBGE and TTBGE) were obtained after 24 h for Amberlyst-15 (~ 20%) and CF_et_-BDS (~ 18%). On the other hand, the process catalyzed by CF_i-bu_-H_2_SO_4_ was the least effective towards the formation of the high-substituted glycerol ethers (as S_DTBGE+TTBGE_ was ~ 9% after 24 h).

**Figure 10:**
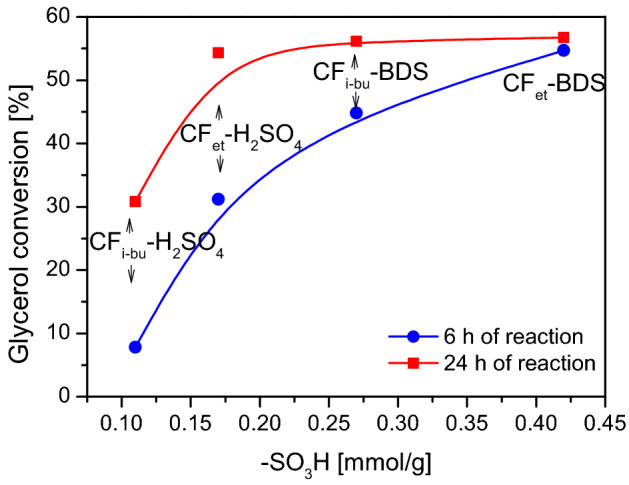
Glycerol conversions versus the concentration of sulfonic groups on CFs for the glycerol etherification with TBA.

A comparison of the presented catalytic data (Figs. 7, 8, 9) with the physicochemical properties of CFs clearly indicates that the catalytic performances of the samples depended mainly on the acidic features of the prepared carbon fibers. Figure 10 depicts the relationship between the conversion of glycerol and the content of -SO_3_H groups for the obtained modified CFs.

As can be seen, there is a clear relationship between glycerol conversions obtained using modified CFs and the content of their surface -SO_3_H groups. Namely, the samples with a higher number of sulfonic groups worked significantly better in the process. This was especially well seen for the shorter reaction time (i.e., 6 h). After 24 h of the process, the differences were not so important, probably due to reaching the reaction equilibrium. It is worth noting that for the glycerol etherification lasting 6 h, CFs showing the lowest concentration of sulfonic groups (i.e., 0.11 mmol/g, see Table 1) gave X_G_ of only about 7.5%, while for CFs with the highest density of -SO_3_H sites (i.e., 0.42 mmol/g), X_G_ was several times higher (over 50%).

**Figure 11:**
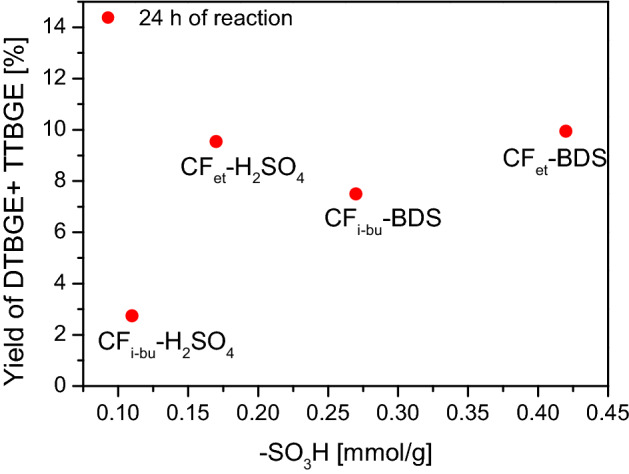
The influence of the content of sulfonic groups on the yield of DTBGE + TTBGE obtained in the etherification of glycerol with TBA.

The most desirable products of glycerol etherification are di- and tri-tert-butyl glycerol ethers (i.e., DTBGE and TTBGE). Figure 11 presents the relationship between the yield of DTBGE + TTBGE obtained in the reaction using the modified CFs and the content of surface -SO_3_H sites.

Generally, the higher the number of sulfonic groups, the higher the combined yield of di- and tri-substituted ethers was obtained. However, the relationship was not as obvious as in the case of X_G_ vs -SO_3_H content (Fig. 10). An interesting sample was CF_et_-H_2_SO_4_. Despite a rather moderate amount of -SO_3_H (i.e., 0.17 mmol/g; see Table 1), CF_et_-H_2_SO_4_ effectively converted MTBGE ethers to DTBGE and TTBGE (see also Fig. 8A). The observed phenomenon was probably related to the relatively high A_tot_ of CF_et_-H_2_SO_4_ (0.39 mmol H^+^/g, Table 1), compared to the other samples, resulting not only from the existence of sulfonic groups but also from the presence of acidic oxygen moieties. Thus, it can be supposed that oxygen functionalities may also have a positive effect on glycerol etherification. Similar conclusions were also drawn for glycerol acetylation performed over functionalized carbonaceous spheres^61^. Generally, it was found there that -SO_3_H groups are active sites of the process; however, oxygen functionalities, even though they do not catalyze the reaction themselves, can improve the reaction by adsorbing the substrates on the catalyst surface, and thus facilitate the contact between reagents and the active sites. The results presented above suggest that such an explanation may also be correct in the case of glycerol etherification over carbon materials. On the other hand, the catalyst porous architecture was probably a parameter of rather secondary importance. For example, the samples modified with BDS showed better catalytic properties than those modified with H_2_SO_4_, even though the BDS-treated CFs presented smaller surface areas (please compare Table 2 and Figs. 7 and 8). This can suggest that S_BET_ is not the critical parameter for obtaining carbon catalysts highly active in glycerol etherification.

## Conclusions

Glycerol etherification leads to valuable glycerol derivatives which may find important applications in many branches of industry. The current research showed that modified carbon fibers obtained from various hydrocarbons by a CCVD process can work as effective catalysts for the reaction between glycerol and tert-butyl alcohol. There were, however, some differences in the susceptibility of CFs to functionalization with sulfonating agents (i.e., concentrated H_2_SO_4_ or benzenediazonium salt generated in situ; BDS) and thus, the sample catalytic activity. The CFs obtained from ethylene were functionalized with -SO_3_H groups more effectively than those prepared from isobutane. Furthermore, a more efficient functionalization method was found to be the modification of CFs with BDS—this approach resulted in introducing significantly higher numbers of -SO_3_H functionalities onto the carbon surface than the reaction of CFs with sulfuric acid. The best catalytic performance in glycerol etherification was observed for CF_et_-BDS which presented nearly 57% glycerol conversion and about 18% combined selectivity to DTBGE and TTBGE within 24 h of the process. These results were generally better than those obtained using Amberlyst-15 under similar conditions. Furthermore, it was found that glycerol conversion is strongly dependent on the contents of -SO_3_H groups. The content of sulfonic functionalities also influenced the observed distribution of products. The presence of oxygen-containing groups also played an important role in the process, acting probably as adsorption sites for the reagents and improving the obtained catalytic results.

## Supplementary Information

Supplementary Information.

## Data Availability

The dataset generated and/or analyzed during the current study is too large to be retained or publicly archived with available resources, thus is not publicly available. Documentation and methods used to support this study are available from the corresponding author upon reasonable request.
