# Supplementary material for: SO3H-functionalized carbon fibers for the catalytic transformation of glycerol to glycerol tert-butyl ethers
Source: Sci Rep. 2023 Jan 11;13:565. doi: 10.1038/s41598-023-27432-7 (PMC9834229; doi:10.1038/s41598-023-27432-7)
Supplement: Supplementary file 1 — Supplementary Information. [file 41598_2023_27432_MOESM1_ESM.docx]

SUPPLEMENTARY MATERIALS

**SO_3_H-functionalized carbon fibers for the catalytic transformation
of glycerol to glycerol tert-butyl ethers**

Karolina Ptaszyńska, Anna Malaika*, Magdalena Kapska, Mieczysław Kozłowski

Faculty of Chemistry, Adam Mickiewicz University

Uniwersytetu Poznańskiego 8, 61-614 Poznań, Poland

Fig. 1SM shows the high-resolution TEM images of different magnifications obtained for CFs produced from isobutane and ethylene.

The presented pictures revealed the partial graphitic structure of CF_i-bu_ and CF_et_, as the ordered orientation of the graphene sheets was visible for part of the produced fibers. In the case of CFs obtained from ethylene, the graphene sheets were arranged parallel to each other and perpendicular to the fiber axis, forming platelet carbon fibers (Fig. 2 E). CFs produced from isobutane also resembled platelet-like structures, however, a slightly lower degree of order than that observed for CF_et_ was noticed, as randomly oriented graphene sheets were also found in the HR-TEM images of CF_i-bu_
(Fig. 1SM B).

*****Corresponding author; e-mail: amalaika@amu.edu.pl; phone: +48 61 829 1800; fax: +48 61 829 1555


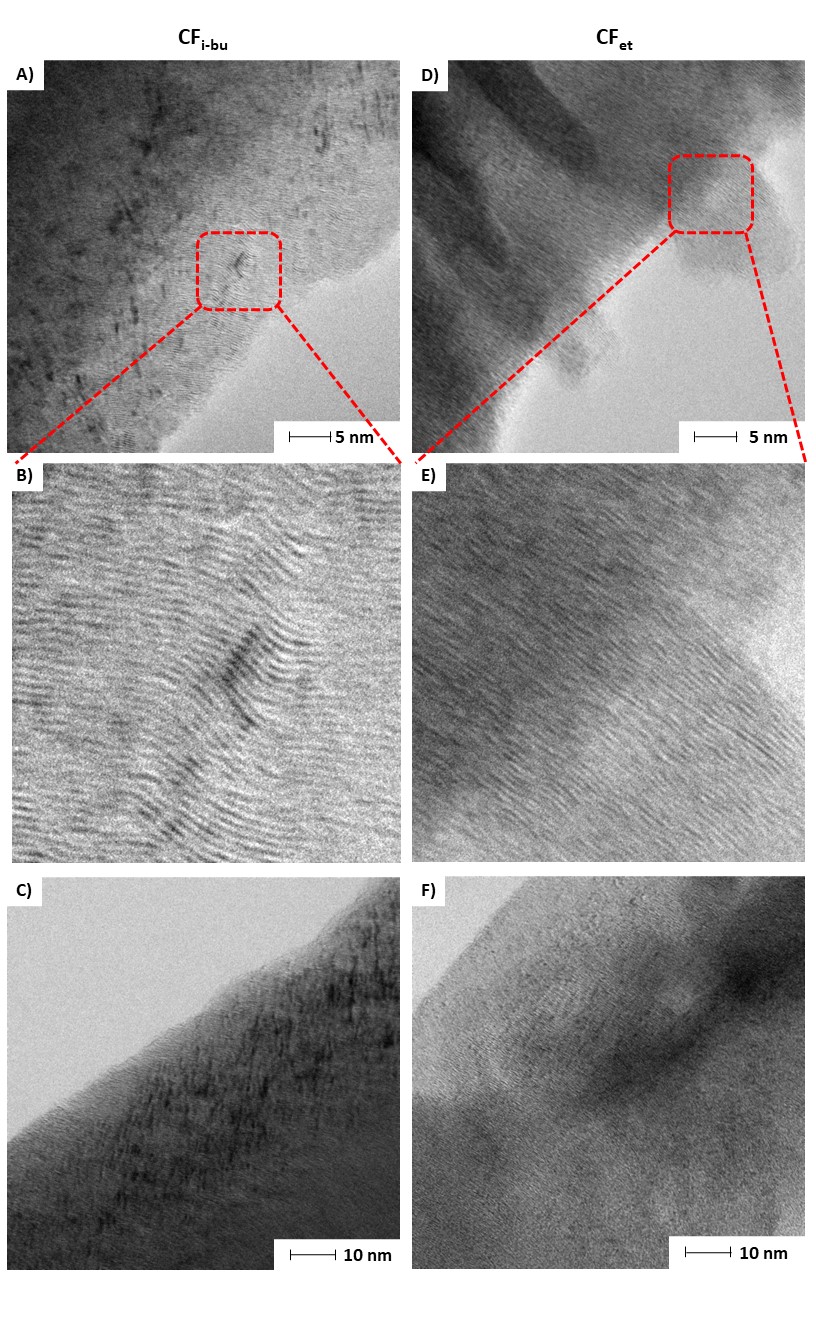


Fig. 1. HR-TEM images of the carbon fibers produced from isobutane (A-C) and ethylene (D-F)
